# Adaptive Serum Biochemistry Responses to Ethanol Administration in a Mouse Model: Implications for Metabolic Regulation Under Analgesia

**DOI:** 10.3390/molecules30224488

**Published:** 2025-11-20

**Authors:** Bożena Witek, Krzysztof Wróbel, Grażyna Świderska-Kołacz, Szymon Zmorzyński, Anna Wojciechowska, Joanna Czerwik-Marcinkowska

**Affiliations:** 1 Institute of Biology, Jan Kochanowski University, 25-406 Kielce, Poland; bozena.witek@ujk.edu.pl (B.W.); grazyna.swiderska-kolacz@ujk.edu.pl (G.Ś.-K.); 2 Expertdent Dental Clinic, 25-371 Kielce, Poland; kontakt@expertdent.pl; 3 Institute of Human Sciences, Academy of Zamość, 22-400 Zamość, Poland; szymon.zmorzynski@akademiazamojska.edu.pl; 4 Department of Geobotany and Landscape Planning, Nicolaus Copernicus University, 87-100 Toruń, Poland; ankawoj@umk.pl

**Keywords:** alcohol, animal model, lipid profile, metabolic markers, pain relief

## Abstract

Analgesia, or reduced pain sensitivity, can result from pharmacological or stress-induced mechanisms, but human studies are limited by complex physiological and psychological variables. This study aimed to evaluate the impact of ethanol consumption on key metabolic markers in two genetically distinct mouse lines selectively bred for pain sensitivity: high analgesia (HA) and low analgesia (LA). Forty-eight male Swiss-Webster mice were randomly assigned to four groups: HA and LA with or without heavy alcohol (ethanol) exposure. Blood serum was analyzed for its lipid profile, enzymatic activity, electrolyte levels and regulatory/energetic compounds. In HA mice, ALAT and AspAT activities and, albumin, creatinine, iron, and potassium levels were elevated, whereas glucose and sodium levels were decreased. LA mice presented increased bilirubin, cholesterol, LDL, HDL, and lipase activity. ChE, LDH, and CK activities differed significantly between the HA and LA groups. Ethanol intake influenced potassium, magnesium, and sodium serum concentrations. Discriminant analysis highlighted distinct biochemical profiles depending on the LA and HA groups. HA mice predominantly exhibit cytolytic liver damage, altered muscle metabolism, and increased iron levels, indicating oxidative stress. HA and LA display distinct adaptive metabolic strategies: protein/muscle and lipid/electrolyte metabolism, respectively. Genetic differences between HA and LA mice determine different metabolic responses to ethanol.

## 1. Introduction

Understanding the interplay between genetic predisposition, pain perception, and the body’s metabolic response remains a critical focus in biomedical research. The present study utilized a transgenerational effect selected mouse model—high analgesia (HA) and low analgesia (LA) lines—which offers a valuable framework for investigating the biological basis of analgesia and variability in pain tolerance. This experimental model helps to overcome the methodological limitations often encountered in human studies, where pain perception is influenced by numerous uncontrolled environmental and psychological factors [1]. The investigation of the effects of ethanol on these lines provides further insight into how external stressors may interact with genetic background to influence both nociceptive and metabolic pathways.

Ethanol is not only one of the most consumed xenobiotics, but also a well-known modulator of pain sensitivity through its interaction with neurochemical systems, including opioidergic and CABAergic signaling [2,3,4]. Moreover, differences in thermoregulatory capacity between HA and LA mice suggest possible differences in metabolic adaptation to biochemical stress. Evaluating key metabolic markers, including lipid profile components, enzymes and electrolytes in conjunction with ethanol intake may offer novel perspectives on the systemic consequences of analgesia-related phenotypes.

Pain, along with its causes, underlying mechanisms, and therapeutic interventions, constitutes a major area of investigation in contemporary medicine. Although nearly universal, pain perception varies significantly among individuals, and is influenced by both genetic and environmental factors. For example, congenital insensitivity to pain, a genetic disorder associated with *SCN9A* gene mutations, is rare [5]. The variation in pain sensitivity across the human population likely reflects differences in neurobiological and metabolic pathways, which are difficult to isolate in clinical settings because of their psychological and physiological complexity [6]. Numerous biochemical markers analyzed in pain research have increased expression in both clinical and preclinical models of nociception [7]. Biomarkers reflecting the presence and nature of pain, including those that differentiate its subtypes and indicate complex interactions between metabolic, immune, and neurobiological systems, have been identified. These results emphasize that the body’s biochemical profile can be a sensitive indicator of pain sensitivity and analgesia modulation. Importantly, the modulation of selected biochemical pathways in animal models—such as the GP130 or TGFβ1 systems—leads to changes in pain intensity, confirming the functional link between metabolic parameters and physiological nociceptive mechanisms [7]. This provides a basis for further research into how differences in genetically determined analgesia (HA vs. LA) may translate into diverse biochemical profiles, especially in the context of an additional metabolic stressor such as ethanol exposure.

Pain responses are essentially stress responses, encompassing not only sensory but also energetic components [8]. Psychological variables often overshadow somatic variables in shaping the pain experience. Consequently, animal models that minimize psychological interference are essential for accurately assessing the biological determinants of pain thresholds [9,10,11]. Alcohol use disorder and chronic pain frequently co-occur and share overlapping neurobiological substrates [12]. Chronic ethanol intake can both induce neuropathic pain and be used as a self-medicated analgesic, creating a feedback loop that reinforces dependence [13,14,15]. Intravenous alcohol has an analgesic effect in cases of acute pain caused by harmful electrical stimulation [16]. In rodent models, alcohol has been shown to have analgesic effects in response to acute thermal stimulation after intraperitoneal administration in hot plate and tail flick tests [17,18].

Accordingly, ethanol was included in this study as a secondary modulating factor in pain perception. Alcohol consumption and chronic pain are characterized by high comorbidity [19]. Compared with the general population, people experiencing chronic pain are more likely to exhibit patterns of problematic alcohol consumption and are up to twice as likely to meet the criteria for alcohol use disorder (AUD) [20]. Alcohol may have analgesic effects [21], its long–term use promotes the progression of chronic pain [20]. In addition, pain may serve as an important motivator for alcohol consumption, as confirmed in both human studies [22] and animal models [23]. Furthermore, alcohol withdrawal after a period of chronic abuse can cause hyperalgesia in people with AUD [24] and alcohol-dependent rats [25]. Consequently, it is postulated that the relationship between pain and alcohol consumption takes the form of positive feedback, leading to mutual intensification of both phenomena over time [20]. Alcohol has analgesic properties, which have been demonstrated in animal models, among other things [17,18,26]. Its well-documented role in altering metabolic and neurological states further supports its use in experimental models exploring analgesia-related traits [27]. Robins and colleagues underscore that despite accumulating knowledge pain tolerance remains difficult to interpret owing to its multifaceted nature [28]. The present study utilized a genetically selected murine model consisting of two lines: high analgesia (HA) and low analgesia (LA) differentiated by stress-induced pain sensitivity over 20 generations. This model facilitates the investigation of gene-environment interactions, particularly how genetic predisposition to pain tolerance influences the physiological response to ethanol.

Previous research has suggested that pain sensitivity and alcohol preference converge on shared neurobiological pathways, especially those involving endogenous opioids [29,30,31]. Thus, HA and LA mice offer an opportunity to examine how these pathways impact metabolic outcomes. Chronic ethanol consumption is known to disrupt lipid metabolism and impair liver and muscle function, as evidenced by changes in gamma-glutamyl transferase (GGT) and creatine kinase (CK) activity [2,32,33]. Chronic ethanol consumption is well documented to disrupt lipid metabolism [34,35,36]. Low-density lipoproteins (LDL) and high-density lipoproteins (HDL) levels determine metabolic disorders, including the potential risk of ethanol-related dyslipidemia [37,38]. These findings may reveal correlations between metabolic variability and differences in pain sensitivity (HA vs. LA). GGT is a biomarker of liver damage and alcohol-induced oxidative stress. Ethanol significantly increases GGT levels, even in the absence of changes in other standard markers of liver damage [39]. Electrolyte imbalances, particularly in sodium and potassium levels, are common in alcohol-related renal dysfunction [40]. The analysis of lipids, liver and muscle enzymes, and serum electrolytes provides a solid foundation for assessing the metabolic response to ethanol administration in an analgesia model. This helps us understand the multifaceted effects of ethanol—from dyslipidemia, through oxidative stress and muscle damage, to electrolyte homeostasis disorders—and the differences between the HA and LA lines.

Differences in analgesia may result from different metabolic profiles and dysregulation of biochemical systems involved in pain modulation, such as the endogenous opioid system, lipid metabolism, or markers of liver and muscle damage. Analysis of biochemical parameters therefore allows us to assess whether HA and LA phenotypes reflect differences not only at the behavioral level, but also at the metabolic level. In this context, the present study aims to improve and extend existing data on the effects of ethanol on nociceptive activity by investigating whether genetically determined differences in analgesia modulate metabolic responses to ethanol. By analyzing serum biomarkers in HA and LA mice, this study aimed to identify biochemical correlates of pain sensitivity in the presence of an additional metabolic stressor. It is hypothesized that ethanol exposure in HA and LA mice will yield distinct biochemical profiles, revealing gene-environment interactions relevant to both pain biology and substance use disorders. To the authors’ knowledge, no similar studies have been conducted on the proposed experimental model.

## 2. Results

Analysis of biochemical parameters revealed that genetically determined analgesia significantly modulates the metabolic response to ethanol. Mice with high analgesia (HA) levels showed numerous and pronounced changes in liver markers, electrolyte balance, and lipid profiles after alcohol administration, whereas in the low analgesia (LA) line, these effects were minimal or absent (Table 1). These findings indicate that ethanol has metabolic sensitivity characteristics in the HA line, leading to greater dysregulation of liver function, ion balance, and protein metabolism. Furthermore, electrolytes and liver parameters were found to be most strongly associated with the analgesic phenotype. The difference in lipid metabolism between the HA and LA lines further confirms the influence of innate pain sensitivity on the metabolic response, with alcohol-induced changes occurring only in the HA line.

### 2.1. Analysis of Liver Parameters in the HA and LA Models

For ALAT and AspAT, the enzyme activities were significantly greater in the HAA group than in the LAA group (Table 1). A direct comparison between HAA and LAA confirmed significantly reduced activities in the LAA group. In contrast, for TBiL, no significant differences were observed between the control and study groups. However, bilirubin concentrations were significantly greater in the LAA group than in the HAA group (Table 1).

### 2.2. Analysis of Enzyme Activities in the HA and LA Models

Statistically significant differences between the control and study groups were found only for ChE (Table 1). When HAA was compared with LAA, significant differences were also observed in the activities of LDH, ChE, and CK (Table 1). In contrast, the results for ALP did not reach statistical significance.

### 2.3. Micro- and Macroelements Concentrations in the HA and LA Models

Iron ions, potassium ions and sodium ions levels differ significantly between the HA and LA genetic groups (Table 1). Higher levels of iron ions and potassium ions were observed in the HAA group. In contrast, a lower sodium level was noted in HAA. Ethanol was found to cause significant changes in potassium, magnesium and sodium levels in both groups (Table 1).

### 2.4. Analysis of Lipid Concentration and Lipase Activity in the HA and LA Models

The concentration of cholesterol was significantly different between the control groups of HA and LA mice (HA/LA—89%) (Table 1). Significant differences were also found between mice receiving ethanol and control mice in the LAA and HAA groups. Higher cholesterol levels—TCHOL and cholesterol transported by LDL and HDL, were observed in the LAA group than in the HAA group (Table 1). In the HDL and LDL cholesterol fractions, significant differences were identified between the HA/LA mouse groups (79% and 41%). Moreover, lipase activity was significantly greater in the LAA group than in the HAA group (Table 1).

### 2.5. Salt Concentrations in the Sera of HA and LA Mice

Significant differences were observed only for chloride levels, both between the study groups and controls as well as between HAA and LAA (Table 1). In contrast, lactate concentrations were not significantly different.

### 2.6. Analysis of Various Compounds

When the controls were compared with the study groups, significant differences in the albumin and creatinine levels were noted (Table 1). Furthermore, the HAA vs. LAA comparison revealed significantly higher albumin and creatinine levels in the HAA group, along with a lower glucose concentration.

### 2.7. Discriminant Analysis

**Figure 1:**
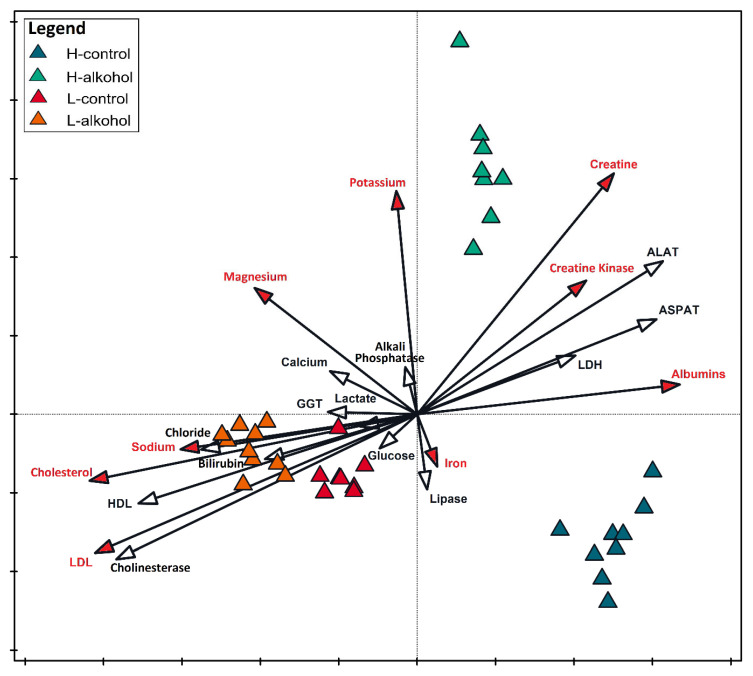
Canonical Variate Analysis for data on the tested biochemical parameters depending on the experimental conditions. The variables that significantly differentiated the research groups are marked with a red vector arrowheads and red names. All the significant variables explained 77.2% of the total variability of the set. Each point represents an individual animal, whereas vectors indicate the contribution and direction of biochemical variables to the overall group separation.

The discriminant analysis is shown in Figure 1 and reveals clear clustering of the four experimental groups. The variant of the experiment in which the low alcohol level was used is distinguished by significantly high levels of sodium, cholesterol and LDL, whereas the high-alcohol variant is distinctly associated with elevated concentrations of creatine, creatine kinase, and potassium. The control conditions of both variants seem to result in a significantly greater level of iron in the serum, and the control for the HA group also included albumins. The H-alcohol (HAA) mice formed a separate cluster in the upper right quadrant of the biplot, closely associated with increased levels of creatine, creatine kinase, ALAT, ASPAT, and potassium, indicating that these parameters most strongly differentiate ethanol-exposed animals with high analgesia. In contrast, the L-control (LAC) and L-alcohol (LAA) groups cluster predominantly on the left side of the plot, a pattern driven by higher concentrations of total HDL, and LDL cholesterol, bilirubin, chlorides, and sodium-suggesting that lipid-related and ionic parameters are key discriminators for the low-analgesia phenotype. The H-control (HAC) group is located in the lower right region and is characterized mainly by higher iron and albumin levels, which contribute to the separation between the baseline HA and LA profiles.

### 2.8. Correlation Analysis

Among the correlations examined, only one had a correlation index value indicating a strong, negative relationship. This one concerns iron levels and is −0.788. A strong, positive correlation was noted for lipase levels. However, neither these nor any of the other correlations examined were statistically significant.

## 3. Discussion

The results revealed significant differences in the TCHOL levels between the HA and LA mouse lines. Ethanol administration led to a significant decrease in TCHOL concentration in LA mice, whereas in HA mice, it caused a statistically significant increase. This divergent response suggests that genetic background related to analgesia sensitivity may influence lipid metabolism under ethanol exposure. Justice and colleagues reported in an animal model that ethanol decreased non-HDL CHOL, TCHOL and liver cholesterol levels [41]. The cholesterol-lowering effects of moderate alcohol consumption are supported by numerous studies, including meta-analyses [42,43,44] although meta-analyses by Matsumoto and colleagues reported no significant associations [45]. Moreover, in our study, notable differences in HDL and LDL cholesterol fractions were observed between the HA and LA groups, with significantly lower levels in HA mice. The consistency of the pattern-across TCHOL, HDL, and LDL suggests that the analgesic phenotype may be associated with distinct lipid regulatory pathways. Ethanol consumption typically increases HDL cholesterol levels in humans [41], and HDL cholesterol was significantly higher in both groups in our study. Importantly, cholesterol not only is a structural component of cell membranes, but also serves as a precursor for steroid hormones, bile acids, and vitamin D, playing a central role in maintaining cellular integrity and metabolic balance [46,47,48]. Its regulation is therefore tightly linked to both endocrine and immune system functions, which may be altered under pain-related stress and alcohol exposure. Although cholesterol is a relatively stable metabolic molecule compared with glucose or fatty acids, recent studies have shown that its levels can still reflect systemic adaptations to chronic stimuli, including stress and nociception [49]. Given these findings, it is plausible that altered cholesterol profiles in HA and LA mice represent part of a broader, genetically driven physiological response to both pain and ethanol. Further investigations into HDL and LDL metabolism in this context are warranted, particularly to understand how these lipid fractions participate in the body’s complex response to prolonged noxious or rewarding stimuli.

In this study, we examined serum liver markers, including the activities of GGT, ALAT, and AspAT, as well as bilirubin levels. GGT constitutes an important element of cell protection from free radicals, so it can play a significant role as a defensive antioxidative factor [50]. GGT play an important role in the generation of free radicals, and some authors consider its activity to be a marker of oxidative stress [37]. This finding suggests that high or low intensity of the response to pain stimuli might be generated depending on the level of those radicals. In the present study, GGT activity did not change significantly on different levels of analgesia (HA or LA). However, we observed higher activities of ALAT and AspAT enzymes in the HA group. Elevated ALAT and AspAT levels with simultaneously reduced bilirubin concentrations may indicate certain specific conditions in the liver. This may indicate mild, early liver damage without bile secretion disorders. An increase in AspAT activity relative to ALAT activity may suggest damage to tissues other than the liver, for example, heart muscle or skeletal muscles [51]. Moreover, in our study we observed decreased levels of ChE in the HAA group, which may indicate liver damage. In contrast, this compound was significantly elevated in the LAA group. In patients with liver disease, opioid metabolism is slowed down, which increases the risk of accumulation and toxic effects. Smaller doses, longer intervals between doses, and close monitoring are recommended [52]. However, studies in humans and animals do not confirm a significant slowing of ethanol metabolism in patients with cirrhosis of the liver compared with healthy individuals [53].

A marker of muscle damage is CK, an enzyme involved in the energetic pathway of phosphorous transformation, as it catalyzes the conversion of a phosphate group from phosphocreatine to ADP [39,54]. The state of acidosis remains in the muscles usually longer than the sensation of pain which might appear rather quickly, even after a few seconds [55]. The results of the present study indicate that HAA mice demonstrated significantly higher levels of CK activity than LAA mice did. Selection for analgesic variability was therefore found to be consequential for CK activity. In the HA and LA groups, no significant effect of ethanol on CK activity was detected. Therefore, LAA mice, whose serum levels of CK activity were lower than those in the HAA group, could use ATP-derived energy more efficiently for immediate analgesic reactions. The results of our experiment suggest that the analgesic effect of alcohol might be related to changes in CK activity, and thus affect the provision of appropriate amounts of energy in the form of ATP, which is crucial for the course of the analgesic reaction [56]. Ethanol has been shown to impact various physiological systems, including muscle function and metabolism [57]. It may disrupt normal muscle recovery and energy metabolism, particularly in the LAA group.

Among the analyzed ions, only sodium and potassium exhibited concentration changes relative to those in control group. Both ions were elevated in the HAA and LAA groups. However, potassium levels were higher in LAA mice than in HAA mice, whereas sodium levels showed the opposite pattern. Potassium is involved in a wide range of physiological activities [58,59]. It is known, indeed, that the regulation of potassium levels in the body takes place at particularly high levels of homeostatic processes [60]. Excretion of a lion’s share of potassium ions with urine and maintaining proper homeostasis are therefore dependent on the active turnover of this ion between the serum and urine. The problem of the regulation of potassium turnover rates upon the administration of substances, such as ethanol, significantly increasing diuresis [61]. Elisaf and colleagues noted that serum potassium concentrations were significantly lower in alcoholic patients than in healthy individuals [62]. These results differ from those obtained in our study suggesting the need for further specialized studies. Sodium is one of the main cations determining the phenomenon of life in terms of general physiological regulation [63]. Most researchers agree with the suggestion that sodium intake in the diet should be limited for preventive purposes, as the absorption of large amounts of sodium might considerably increase the risk of cardiovascular diseases. This limitation also seems to have some effect on the activity of catecholamines and the level of serum lipids [64,65]. The restraint on sodium consumption is considered positive, in contrast to the negative effects of its markedly high levels [66]. A low concentration of sodium in the blood might contribute to reduced intensity of oxidative stress [67]. In our study, we confirmed that sodium—as an indicator of the genetic variability of mice between high analgesia (HA) and low analgesia (LA)—in mice, significantly changed (HA/LA—99.7%). Significant changes in sodium concentration were found in relation to ethanol consumption, both in the AH group and the LA group. Sodium and chloride homeostasis disorders may predict treatment outcomes in patients with advanced chronic liver disease [68]. Hypochloremia is associated with increased mortality in clinically stable and critically ill patients with cirrhosis, which can be caused by chronic alcohol consumption [46]. In our study, higher chloride concentrations were observed in both the HA and LA groups. Elevated serum chloride, or hyperchloremia, can occur in hyperchloremic metabolic acidosis, renal failure—particularly when the kidneys’ ability to excrete chloride is impaired and severe liver diseases, such as cirrhosis or inflammation. Elevated CREA levels in the HAA group may suggest the occurrence of kidney damage. Abnormal glucose and albumin concentrations could also indicate liver dysfunction in this group.

The primary effects of ethanol are exerted at the neurological level [69]. The acute effects of alcohol are mediated by interactions with amino acid neurotransmitters, as well as by parallel changes in amines such as noradrenaline, dopamine, and serotonin [70]. They include enhancing GABAergic inhibition, inhibiting NMDA-mediated excitation and modulating endogenous opioid and dopaminergic pathways [71]. Studies have shown that alcohol increases glycine receptor function in laboratory preparations [72]. The sedative effects of alcohol may be produced by neurotransmitter systems interacting with each other. One example of this phenomenon occurs in Purkinje cells, which are a type of neuron found in the cerebellum. In these cells, increased activation of the GABA_A receptor induced by alcohol occurs only with the concurrent activation of certain norepinephrine receptors. Norepinephrine is a neurotransmitter with many regulatory functions [73]. Systemic physiological responses that can be detected in serum biochemical parameters are triggered by these central effects. The elevated CK observed in HAA mice may be explained by increased muscle relaxation and altered neuromuscular transmission, as reduced motor control and ethanol-induced myocyte stress can release CK into the circulation [74,75]. Similarly, ethanol-induced autonomic dysregulation affects hepatic blood flow, oxidative stress and sympathetic activity, leading to shifts in ALAT, AspAT, bilirubin and albumin concentrations [76]. Changes in sodium and potassium levels may reflect altered neuronal excitability and alcohol-related modulation of ion channels that maintain the membrane potential [77]. Finally, ethanol-driven changes in lipid metabolism are partially regulated by central control of energy balance and stress pathways, including the hypothalamic–pituitary–adrenal axis, which interacts with both nociceptive processing and metabolic homeostasis [78]. Therefore, although ethanol primarily affects the nervous system, its systemic effects are reflected in serum biochemistry, providing an indirect indication of genotype-dependent differences in the physiological response to alcohol

This study has several limitations that should be acknowledged. First, while biochemical parameters were comprehensively analyzed, the expression of protein-coding genes was not assessed. Transcriptomic or proteomic profiling would provide a broader perspective on the molecular pathways underlying the distinct metabolic responses observed in HAA and LAA mice. Second, liver morphology was not examined. Histopathological analysis could have strengthened the interpretation of differences in hepatocellular injury (elevated ALAT and AspAT in HAA vs. altered bilirubin metabolism in LAA). Third, the hepatocyte phenotype was not validated via flow cytometry, which would allow confirmation of the cell subpopulations potentially responsible for the divergent biochemical responses. Fourth, ultrastructural analysis of hepatocytes via electron microscopy was not performed, thereby limiting the evaluation of intracellular changes such as mitochondrial alterations, cytosolic damage, or nuclear abnormalities that might accompany the observed metabolic differences. Another limitation is that only the serum parameters were evaluated. Although they provide valuable insight, they do not fully capture tissue-specific changes (e.g., direct muscle injury reflected in CK elevation, or lipid accumulation in hepatocytes linked to cholesterol imbalance). The relatively small sample size also reduces the statistical power of the findings and limits the ability to generalize the results. Despite these limitations, this study represents the first comprehensive biochemical characterization of HAA and LAA models under ethanol exposure, offering a valuable framework for understanding how genetic background modulates liver injury, lipid metabolism, and systemic homeostasis.

Although ethanol caused clear and genotype-dependent changes in the metabolic parameters studied, this does not explain the differences in sensitivity to analgesics, as none of the metabolites measured were significantly correlated with analgesia-related traits.

## 4. Materials and Methods

This study was conducted at Jan Kochanowski University in Kielce (Poland) and the Institute of Genetics and Animal Biotechnology of the Polish Academy of Sciences in Jastrzębiec (Poland). All procedures were carried out in accordance with the relevant institutional and national guidelines for animal care and use and were approved by the Świętokrzyska Chamber of Physicians and Dentists in Kielce (approval no. 3/B/2021).

### 4.1. Animal Test Subjects and Tissue Processing

Forty-eight male Swiss-Webster mice aged 8 weeks with an initial body weight of 25.0 ± 2.0 g, were group-housed and obtained from the Institute of Genetic and Animal Biotechnology of the Polish Academy of Sciences (Jastrzębiec, Poland). They were acclimated for 6 days, and then single-housed, and testing started 3 days later. The mice remained single-housed throughout the duration of the experiment. The mice were maintained under standard laboratory conditions at a constant temperature of 21–22 °C and a relative humidity of 50–60% under a 12 h light/dark cycle (12L–12D). All the mice were placed in standard, polycarbonate cages (252 × 167 × 140 mm) covered with stainless lids with a built-in feed hopper. Food (Field Station Łomna-Las, near Warsaw, Poland) and water (reverse osmosis purified) were available ad libitum except during affective and locomotor testing. All the mice were given standard food containing (on a per weight basis) 15.63% protein and 14.04 MJ/kg energy. For free-choice drinking model, the mice were presented with 2 bottles during the ethanol access period, one containing diluted ethanol (e.g., 8% *v*/*v*) and the other containing water, which provided a choice to the mice. The amount of ethanol solution or water consumed was determined by comparing the amount of fluid in the bottles before and after the access period. Upon reaching sexual maturity (6 weeks), experimental mice were selected at random from the entire population and subjected to a two-way selection with the aim of establishing an animal model of different sensitivities to pain, that is individuals with HA and LA. Selection was continued for 20 generations through mating HA males and females, which demonstrated low susceptibility and thus high tolerance to acute pain induced by thermal factors, as did LA males and females, which demonstrated high susceptibility and thus low tolerance to pain stimuli.

### 4.2. Pain Sensitivity Testing

The standard hot plate (HP) test was used to determine pain thresholds and the experimental mice were classified into HA and LA groups. The test involved placing a mouse onto a copper testing plate heated to 56 °C and measuring the pain response latency, that is the time span between placing the mouse on the hot plate surface and the occurrence of characteristic reflexes of licking or leg flicking. Male and female mice that were maintained on the hot plate for approximately 10 s were selected for further stages of the study. These mice were classified as highly resistant to pain stimuli and were thus included in the HA group. The mice that managed to endure only 2–3 s on the heated plate were classified into the LA group. HA males and females were mated to produce offspring leading to future generations with the HA genotype, whereas LA males and females were mated to produce future generations with the LA genotype [79]. At the same time, a control line of mice was bred, and not subjected to selection [80].

### 4.3. Experimental Procedure

The mice were divided randomly into 4 groups: 2 experimental groups and 2 control groups (12 mice per group):

a/ experimental group—HA-treated mice;
b/ experimental group—LA-treated mice;
c/ control group—mice showing HA;
d/ control group—mice showing LA.

The procedural details for this model are described below:

- 60 mL clear graduated bottles (Qorpak, Clinton, PA, USA, clear graduated medium round bottles, GLC-01472).
- Rubber rodent water bottle nozzles (alternative design, neoprene stopper, and straight open sipper tube).
- Standard drinking bottle for water (Tecniplast, Buguggiate, Italy, animal water bottles, 600 mL).

The mice (N = 48) were allowed limited access to alcohol (2 h/day at 10.00–11.00 am and 7.00–8.00 pm for a period of 21 days) and were allowed to freely choice (8% *v*/*v* ethanol vs. water) via the drinking procedure starting 3 h into the dark phase of the circadian cycle. At the conclusion of the experiment, the mice were subjected to anesthesia (Morbital, Biowet Puławy, Puławy, Poland) and then decapitated. Blood was collected into vials containing heparin and centrifuged (MPW-351 R, MPW Med Instruments, Warsaw, Poland) at 3000 rotations per minute to obtain serum. An 8% ethanol solution is commonly used in rodent studies because it strikes a balance between palatability, voluntary consumption, and pharmacological relevance. At this concentration, mice are more likely to consume ethanol voluntarily without the need for sweeteners or forced administration, thereby preserving the naturalistic modeling of alcohol intake [81]. Lower concentrations may not produce sufficient blood ethanol concentrations to model human alcohol consumption and its physiological effects, whereas higher concentrations can reduce intake due to aversive taste or toxic effects.

Additionally, 8% ethanol approximates the alcohol content of commonly consumed beverages such as wine or strong beer, making it a relevant translational model. This concentration also reliably produces measurable changes in behavior, metabolism, and organ function, allowing researchers to assess both the acute and chronic consequences of alcohol use in a controlled setting [82]. Using a standard concentration across studies also facilitates reproducibility and comparison between experiments.

### 4.4. Serum Parameters

In blood serum, the following parameters were determined: concentration of metabolic indices—total cholesterol (TCHOL), HDL and LDL fraction cholesterol; activity of enzymes: gamma-glutamyl–transferase (GGT) (EC 2.3.2.2) and creatine kinase (CK) (EC 2.7.3.2); and levels of potassium and sodium ions [83]. Laboratory analysis of metabolic indices, enzyme activity and ion levels were performed in accordance with dedicated research methodology [83], using a COBAS INTEGRA 400 plus analyzer (Roche Diagnostics Ltd., Rotkreuz, Switzerland), as well as specific analytical kits appropriate for the determination of individual parameters.

### 4.5. Albumin (ALB)

The concentration was measured via a colorimetric test, the so-called endpoint method, in which the increase in absorbance at a wavelength of λ = 583 nm was used. The results are shown in g/L.

### 4.6. Total Protein (TP)

The level was measured via a colorimetric method, which the increase in absorbance at a wavelength of λ = 552 nm was used. The results are shown in g/L.

### 4.7. Total Bilirubin (TBIL)

The concentration was measured via a colorimetric method, using the increase in absorbance at a wavelength of λ = 552 nm was used. The results are shown in μmol/L.

### 4.8. Total Cholesterol (TCHOL)

The concentration was measured via an enzymatic-colorimetric method. The intensity of the color of the final product (quinone imine), which is directly proportional to the cholesterol concentration, was assessed. The determinations were made by measuring the increase in absorbance at a wavelength of λ = 512 nm. The results are shown in mmol/L.

### 4.9. HDL Cholesterol (HDL CHOL)

A homogeneous enzymatic method was used to determine the concentration using cholesterol esterase and cholesterol oxidase. The intensity of the color of the red dye (quinone imine) was assessed. The intensity of the color is directly proportional to the cholesterol concentration of the HLD fraction. The determinations were made by measuring the increase in absorbance at a wavelength of λ = 583 nm. The results are shown in mmol/L.

### 4.10. LDL Cholesterol (LDL CHOL)

The concentration was determined via a homogeneous enzymatic method via selective micellar solubility of LDL cholesterol particles using a non-ionic detergent and the interaction of the sugar component with lipoproteins (VLDL). The intensity of the quinone imine dye formed is directly proportional to the concentration of LDL cholesterol. The determinations were made by measuring the increase in absorbance at a wavelength of λ = 583 nm. The results are shown in mmol/L.

### 4.11. Glucose (GLU)

The concentration was determined via a reference-enzymatic method involving hexokinase and NADPH. The concentration of NADPH is directly proportional to the glucose concentration. The determinations were made by measuring the absorbance at a wavelength of λ = 340 nm. The results are shown in mmol/L.

### 4.12. Creatinine (CREA)

The concentration was determined via the modified Jaffe method, without deproteinization. This method uses the ability of creatinine to bind with picrates in an alkaline environment, resulting in the formation of a yellow-red complex. The amount of the complex formed is proportional to the creatinine content. The absorbance was measured at a wavelength of λ = 512 nm. The results are shown in μmol/L.

### 4.13. Triacylglycerols (TAG)

The concentration was determined via an enzymatic-colorimetric method. The intensity of the red color of the final product (quinone imine) was assessed. The color intensity is directly proportional to the triacylglycerol concentration. The results are shown in mmol/L.

### 4.14. Enzymes

- Alanine aminotransferase (ALAT, EC 2.6.1.2)

The activity was measured via a spectrophotometric method with pyridoxal phosphate according to the IFCC method, which uses the kinetic reaction of the decrease in absorbance at a wavelength of λ = 340 nm. The results are shown in U/L.

- Aspartate aminotransferase (AspAT, EC 2.6.1.1)

The activity was measured via a spectrophotometric method according to the IFCC system without pyridoxal phosphate. The absorbance decrease reaction was tested at a wavelength of λ = 340 nm. The results are shown in U/L.

- Cholinesterase (ChE, EC 3.1.1.8)

Activity determination was performed using thiocholine butyrate as a substrate. The absorbance was read at a wavelength of 405 nm or 415 nm. The results are shown in U/L.

- Lactate dehydrogenase (LDH, EC 1.1.1.27)

The activity was determined spectrophotometrically by measuring the increase in absorbance at a wavelength of λ = 340 nm. The results are shown in U/L.

- Alkaline phosphatase (ALP, EC 3.1.3.1)

Activity determination was performed calorimetrically, which involves the decomposition of a synthetic substrate (4-nitrophenyl phosphate) by alkaline phosphatase. The amount of released 4-nitrophenol is directly proportional to the enzyme activity. The determination was performed by measuring the increase in absorbance at a wavelength of λ = 409 nm. The results are shown in U/L.

- Gamma glutamyl transferase (GGT, EC 2.3.2.2)

The activity was determined via an enzymatic-colorimetric method. The purpose of this method is to measure the amount of released 5-amino-2-nitrobenzoate, which is proportional to the enzyme activity. The determination was performed by measuring the increase in absorbance at a wavelength of λ = 409 nm. The results are shown in U/L.

- Creatine kinase (CK, EC 2.7.3.2)

The method used for the determination was in accordance with the recommendations of the IFCC, SFBC, SCE and DGKC. The activity was determined via the degree of NADPH oxidation, which is directly proportional to the catalytic activity of the enzyme. The determination was performed by measuring the increase in absorbance at a wavelength of λ = 340 nm. The results are shown in U/L.

- Lipase (EC, EC 3.1.1.3)

The activity was determined via a colorimetric method, in which 1,2-o-dilauryl-rac-glycero-3-glutaric acid 6-methylresorufin ester, which is specifically degraded by lipase in the presence of colipase and bile acids, was used as a substrate. The resulting methylresorufin is red and its absorbance is directly proportional to the lipase activity at a wavelength of λ = 583 nm. The results are shown in U/L.

### 4.15. Electrolytes

- Chlorides, sodium and potassium ions

Electrolyte analysis (Cl, Na, and K) was performed via the potentiometric method of ion-selective electrodes [83]. The results are shown in mmol/L.

- Phosphates

Concentration determination was performed via the end-point method with a blank test. The concentration of phosphomolybdate formed is directly proportional to the concentration of inorganic phosphate. The determination was performed by measuring the increase in absorbance at a wavelength of λ = 340 nm. The results are shown in mmol/L.

- Magnesium ions

The colorimetric method with chlorophosphonazo III (CPZ III) was used to determine the concentration, that binds magnesium, increasing the absorbance at a wavelength of =659 nm. The difference between the absorbance of the magnesium-CPZ III complex and the complex after reaction with EDTA is the absorbance due to magnesium ions. The results are shown in mmol/L.

- Lactate

The concentration was determined via an enzymatic-colorimetric method. The intensity of the color formed was directly proportional to the concentration of L-lactate in the sample. The determination was performed by measuring the increase in absorbance at a wavelength of λ = 552 nm. The results are shown in mmol/L.

- Calcium ions

The concentration was determined via a method which o-cresolphthalein was used as a substrate, in which the increase in absorbance at a wavelength of λ = 552 nm was measured. The results are shown in mmol/L.

- Iron ions

The concentration was determined via the colorimetric method with ferrozine, without deproteinization, by measuring the increase in absorbance at a wavelength of λ = 552 nm, and measuring the increase in absorbance at a wavelength of 552 nm. The results are shown in μmol/L.

### 4.16. Statistical Analyses

For each of the biochemical parameters studied, the Kruskal–Wallis test was performed, the aim of which was to indicate the significance of differences between the experimental variants. This test was chosen because of the small number of groups and the unequal number of elements in the groups. Dunn’s test was used as a post hoc test. These analyses were performed via the PAST 4.16c program [84]. Canonical Variate Analysis (CVA) was performed to show how the biochemical parameters studied differentiate the experimental variants used. This is a type of discriminant analysis, during which a Monte Carlo permutation test was performed to determine the statistical significance and variability of the biochemical variables studied. The result of the analysis is an ordination diagram, in which the experimental variants are marked with symbols, and the studied variables are marked with vectors. This analysis was performed in the Canoco 5.0 program [85]. Correlation analysis was performed for each of the biochemical parameters studied. Spearman’s correlation coefficient was calculated, which indicates the relationship between the values of a given parameter under low and high alcohol levels. The statistical significance of the correlation coefficient was also reported. These analyses were performed via the PAST 4.16c program [84].

## 5. Conclusions

This study employed a transgenerational effect selected murine model, comprising HA and LA lines as a robust framework for investigating the biological underpinnings of pain tolerance and analgesia. By examining the effects of ethanol in these distinct genotypes, we gained insights into how environmental stressors interact with genetic predispositions to influence nociceptive and metabolic responses. On the basis of results obtained, the following conclusions can be drawn: (I) the mechanisms of liver damage may differ between the HAA and LAA groups—cytolytic mechanisms predominate in HAA, whereas bilirubin metabolism disorders predominate in LAA; (II) in addition to hepatocyte damage, HAA also causes changes in muscle metabolism, while LAA is characterized by a lower severity of these effects; (III) ethanol significantly disrupts electrolyte homeostasis, particularly Na^+^ and K^+^. Higher iron levels in HAA may be associated with increased oxidative stress (iron catalyzes Fenton reactions); (IV) in the LAA group, alcohol induces hyperlipidemia and increased lipase activity, which may indicate increased lipid metabolism and metabolic stress. In HAA, however, these mechanisms are less pronounced; (V) different adaptive strategies of the body to ethanol metabolism were observed—HAA is more catabolic, and LAA is more lipidemic. HAA and LAA differ in their metabolic strategies for adapting to alcohol—HAA involves protein/muscle metabolism to a greater extent, while LAA involves lipid and electrolyte metabolism. Genetic differences between HA and LA mice determine different metabolic responses to ethanol. This approach may contribute to a more comprehensive understanding of gene-environment interactions relevant to pain modulation, metabolic health, and substance use vulnerability.

## Figures and Tables

**Table 1:** Mean values of the tested biochemical parameters (±SE) in the tested experimental groups. In the case of parameters for which the differences were statistically significant (Kruskal–Wallis test, ns—not significant, *p* > 0.05), the post hoc test (Dunn’s test) was applied. The results of the post hoc test are indicated by different lowercase letters.

Group	Parameter	H-Control	H-Alcohol	L-Control	L-Alcohol	*p*-Value
Liver parameters	ALAT	66.5 ^a^ ± 7.58	73.0 ^a^ ± 10.3	31.4 ^b^ ± 2.02	30.2 ^b^ ± 1.35	*p* < 0.001
	AspAT	488 ^a^ ± 47.2	541 ^a^ ± 47.4	349 ^b^ ± 32.2	306 ^b^ ± 19.6	*p* < 0.001
	Bilirubin	1.16 ± 0.10	1.12 ± 0.11	1.57 ± 0.17	1.46 ± 0.15	ns
	GGT	0.45 ± 0.14	0.58 ± 0.14	0.81 ± 0.31	0.69 ± 0.20	ns
Enzymes	LDH	3143 ± 1106	3424 ± 1188	1560 ± 158	1200 ± 0.25	ns
	Alkaline phosphatase	90.6 ± 3.92	93.4 ± 4.89	90.2 ± 5.42	92.9 ± 3.53	ns
	Cholinesterase	3678 ^a^ ± 178	3546 ^a^ ± 53.9	4745 ^b^ ± 183	4906 ^b^ ± 105	*p* < 0.001
	Creatine Kinase	7984 ± 1449	9695 ± 981	6318 ± 965	5040 ± 896	ns
Micro and macro elements	Iron	34.9 ± 0.82	36 ± 1.79	35.86 ± 1.66	34.2 ± 0.83	ns
	Potassium ions	8.83 ^a^ ± 0.06	9.62 ^b^ ± 0.18	8.94 ^a,b^ ±0.16	9.14 ^a,b^ ± 0.13	*p* < 0.01
	Calcium ions	2.28 ± 0.04	2.33 ± 0.03	2.24 ± 0.06	2.35 ± 0.03	ns
	Magnesium ions	0.97 ^a^ ± 0.01	1.06 ^b^ ± 0.02	1.06 ^b^ ± 0.02	1.07 ^b^ ± 0.02	*p* < 0.01
	Sodium ions	147 ^a^ ± 0.56	148 ^a^ ± 0.85	147 ^a^ ± 0.52	151 ^b^ ± 0.54	*p* < 0.01
Fatty acids	Cholesterol	1.98 ^a^ ± 0.03	2.12 ^a^ ± 0.04	2.72 ^b^ ± 0.08	2.85 ^b^ ± 0.12	*p* < 0.001
	LDL CHOL	0.09 ^a^ ± 0.007	0.08 ^a^ ± 0.009	0.25 ^b^ ± 0.02	0.28 ^b^ ± 0.02	*p* < 0.001
	HDL CHOL	1.71 ^a^ ± 0.03	1.74 ^a^ ± 0.04	2.16 ^b^ ± 0.11	2.31 ^b^ ± 0.11	*p* < 0.001
	Lipase	28.3 ± 0.96	26.6 ± 1.06	29.8 ± 2.67	28.3 ± 0.63	ns
Salts	Chlorides	115 ^a^ ± 0.54	116 ^a^ ± 0.61	116 ^a^ ± 0.52	117 ^b^ ± 0.34	*p* < 0.01
	Lactate	8.63 ± 0.48	9.16 ± 0.73	8.79 ± 0.37	9.69 ± 0.55	ns
Building, regulatory and energetic compounds	Albumins	31.1 ^a^ ± 0.37	30.7 ^a^ ± 0.59	28.9 ^b^ ± 0.49	28.5 ^b^ ± 0.35	*p* < 0.001
	GLU	7.30 ± 0.37	6.92 ± 0.30	8.09 ± 0.22	7.23 ± 0.30	ns
	CREA	11.0 ^a^ ± 0.57	13.3 ^a^ ± 0.49	9.04 ^a,b^ ± 0.52	8.86 ^b^ ± 0.17	*p* < 0.001

H—High; L—low.

## Data Availability

The original contributions presented in this study are included in the article. Further inquiries can be directed to the corresponding author.

## Abbreviations

The following abbreviations are used in this manuscript:

ALAT, alanine aminotransferase; ALB, albumin; ALP, alkaline phosphatase; AspAT, aspartate aminotransferase; BEC, blood ethanol concentration; ChE, cholinesterase; CK, creatine kinase; CREA, creatinine; EC, lipase; GGT, gamma-glutamyl–transferase; GLU, glucose; HA, high analgesia; HAA, high analgesia alcohol; HAC, high analgesia control; HDL CHOL, high density lipoproteins cholesterol; LA, low analgesia; LAA, low analgesia alcohol; LAC, low analgesia control; LDH, lactate dehydrogenase; LDL CHOL, low density lipoproteins cholesterol; TAG, triacylglycerols; TBIL, total bilirubin; TCHOL, total cholesterol; TP, total protein.
